# Compound amino acid combined with high-dose vitamin B6 attenuate traumatic coagulopathy via inhibiting inflammation by HMGB1/TLR4/NF-κB pathway

**DOI:** 10.1186/s12950-020-00258-0

**Published:** 2020-08-28

**Authors:** Shi-Jian Yi, Yang Wu, Lan-Lan Li, Qian-Kun Liang, Yue Xiao

**Affiliations:** 1 Department of General Surgery, Shenzhen University General Hospital, No.1098, Xueyuan Avenue, Nanshan District, Shenzhen, 518055 Guangdong Province People’s Republic of China; 2 Department of Infection Control, Shenzhen Fuyong People’s Hospital, Shenzhen, 518103 People’s Republic of China; 3 Department of Outpatient, Shenzhen University General Hospital, Shenzhen, 518055 People’s Republic of China

**Keywords:** Compound amino acid, Vitamin B6, Traumatic coagulopathy, Inflammatory response, HMGB1/TLR4/NF-κB signaling pathway

## Abstract

**Background:**

Traumatic coagulopathy (TC) arises primarily from coagulation system failure to maintain adequate hemostasis after serious blood loss or trauma. Circulatory homeostasis restoration is the mainstay of the therapeutic approach to TC, but the effects are significantly inhibited by coagulopathy.

**Objective:**

To identify the therapeutic effects and underlying mechanism of compound amino acid (CAA) combined with high-dosage of vitamin B6 (VB6) on TC.

**Methods:**

Rabbit traumatic model and cellular model were used to evaluate the effect of CAA combined with high-dosage of VB6 in TC. Blood concentrations of AST and ALT were measured using the Vitros 250 device while blood APTT, PT and TT concentrations were measured using commercial diagnostics kits. Furthermore, qRT-PCR, ELISA and Western blotting were used to determine the expression of clotting factor (II, VII, IX, X and XI), inflammatory factors (TNF-α, IL-6 and IL-1β) and HMGB1/TLR4/NF-κB signaling-related proteins, respectively.

**Results:**

In the rabbit traumatic model, CAA combined with high-dosage of VB6 therapy inhibited the high expression of AST and ALT, but increased the expression of coagulation factors. Additionally, in both the rabbit trauma model and cellular injury model, CAA combined with high-dosage of VB6 inhibited the expression of inflammatory factors (IL-6, TNF-α and IL-1β) and proteins (HMGB1, TLR4 and p-p65) in HMGB1/TLR4/NF-κB pathway. Most importantly, over-expression of HMGB1 reversed the effect of CAA and VB6 in HUVECs and EA.hy926 cells injury model.

**Conclusion:**

CAA combined with high-dosage of VB6 alleviated TC and inhibited the expression and secretion of inflammatory factors by inhibiting HMGB1-mediated TLR4/NF-κB pathway.

## Background

Traumatic coagulopathy (TC), which arises from intrinsic dysregulation of blood coagulation system, leads to blood loss, shock and eventually death among most severely injured patients [1]. Although the physiological environment in which TC originates from is not fully elucidated, it is reported that inflammation, anticoagulant dysfunction as well as cellular dysfunction from any other pathogenesis contribute to TC [2, 3]. However, TC is the most frequent cause of mortality in trauma patients and accounts for 40% of deaths [4]. Therefore, it is urgent to find novel therapies for the treatment of TC.

Vitamin B6 (VB6) broadly refers to six pyridine vitamins: pyridodol (PN), pyridoamine (PM), pyridoaldehyde (PL) and their 5′-phosphorylated forms (PNP, PMP, and PLP) [5]. VB6 is involved in a number of biochemical reactions as an enzyme cofactor [6]. Most importantly, it is essential in amino acid metabolism where it acts as a growth factor and is believed to be involved in alleviating coagulation problems [7, 8]. VB6 has been reported to have significant antioxidant and anti-inflammatory biological activities due to its 3-hydroxyl radical of pyridine [9–11]. In the past few years, a clear and strong relationship between VB6 and inflammation has been proven, but the mechanism of this relationship is not clear. During inflammation, the content of PLP is inversely related to the severity of the disease and inflammation markers including IL-6, TNF-α and IL-1β [12, 13]. 20AA Compound Amino Acid (CAA) Injection (Fengnuoan, 500 mL, Cisen Pharmaceutical Co., Ltd., China), a sterilized aqueous solution prepared from 20 kinds of amino acids, is mainly used to prevent and treat hepatic encephalopathy, liver disease, or intravenous nutrition in the acute phase of hepatic encephalopathy, and provides a substrate for body metabolism. CAA can not only correct the imbalance of branched-chain amino acids and aromatic amino acids, but also inhibit the formation of pseudo-neurotransmitters in the brain and improve hepatic encephalopathy [14]. It was reported that CAA combined with high-dose VB6 could relieve hemorrhage of patients with coagulopathy after severe trauma [14]. Additionally, previous study indicated that leucine-rich essential amino acids can stimulate mammalian targets of rapamycin (mTOR), and overexpression of mTOR can reduce the inflammation of myocardial cells and prevent cardiac dysfunction by inhibiting the inflammatory response caused by IL-6 [15]. Proline protects the liver against inflammatory injury and liver failure by activating the IL-6/STAT3 survival signaling pathway. Furthermore, it’s well established that the correlation between fibrinogen’s oxidative function to a specific methionine and post-traumatic clot formation was confirmed [16]. However, the specific function of CAA combined with VB6 in the treatment of coagulation problems was rarely reported.

The high-mobility group box 1 (HMGB1) is secreted by platelets and up-regulated under abnormal coagulation, sepsis, disseminated intravascular coagulation as well as trauma [17]. Reportedly, HMGB1 acts as a pro-inflammatory cytokine, which promotes cells migration and affects cell proliferation as well as activates the inflammatory condition [18]. In addition, HMGB1 activates inflammatory responses by stimulating such receptors as toll like receptor 4 (TLR4) [19]. On the other hand, TLR4 is an important activator and aggregator of platelets’ initiation of thrombus formation in hemorrhagic shock and leading to hemorrhage cessation [20]. Studies have shown that the HMGB1/TLR4/NF-κB signaling pathway is involved in multiple inflammatory responses [21]. However, the mechanisms of the HMGB1/TLR4/NF-κB signaling pathway in TC are not yet fully understood.

The current study evaluated the effect and underlying mechanism of the CAA and VB6 combination therapy in the treatment of TC. Most importantly, administration of CAA and high-dosage of VB6 relieved TC in rabbits and inhibited endothelial cell inflammatory response in HUVEC and EA.hy926 cell lines. Followed up mechanistic studies revealed the inhibition of endothelial cell inflammation by inhibiting HMGB1/TLR4/NF-κB signaling pathway, suggesting CAA and VB6 alleviate TC through such pathway. Our results indicated that the CAA combined with high-dosage of VB6 may improve TC and provide a novel therapy for TC treatment.

## Results

### CAA combined with high-dosage VB6 relieved TC in rabbits

**Fig. 1:**
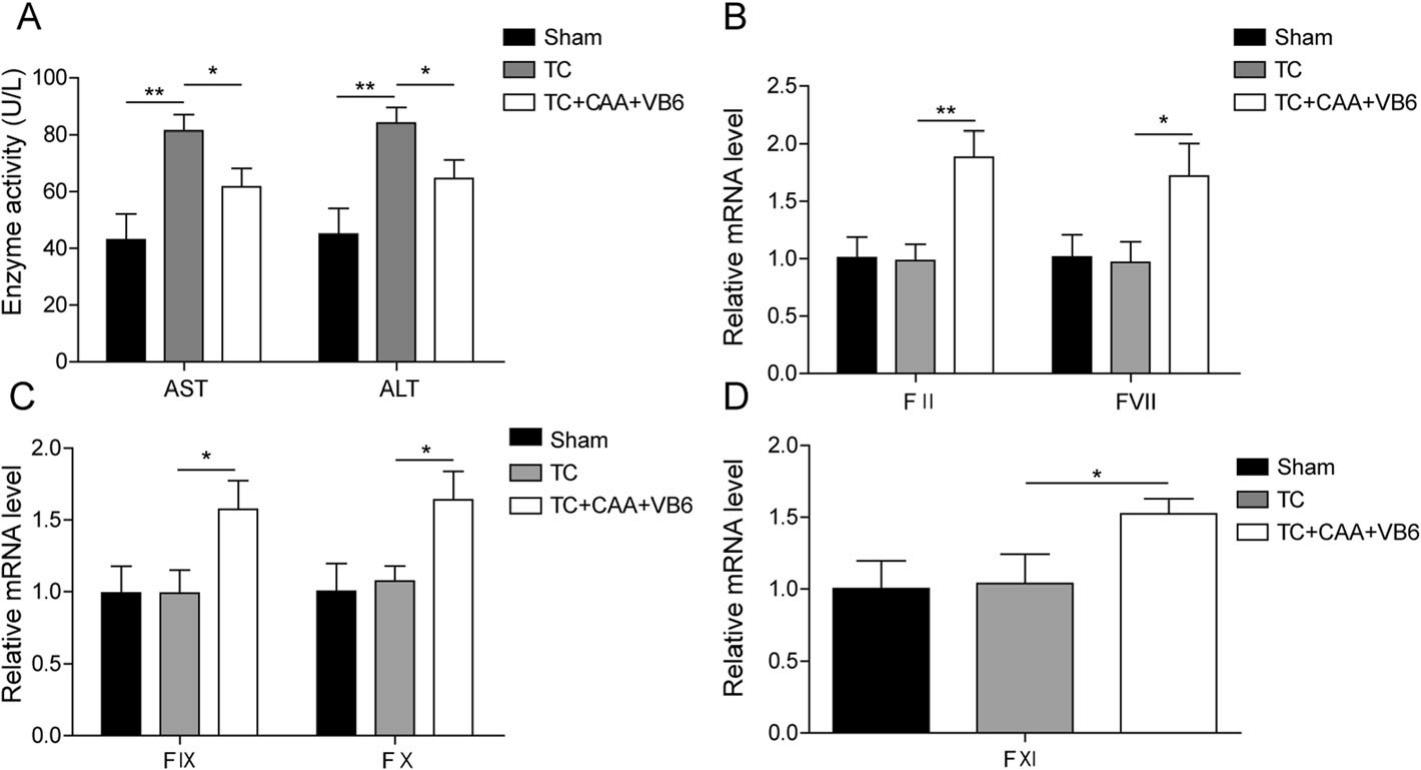
Combination of CAA with high-dosage of VB6 relieved TC in rabbits. Each group used 5 New Zealand white rabbits. **a** AST and ALT levels in rabbit serum from different groups by AST and ALT assay. **b-d** Clotting factors (II, VII, IX, X and XI) expression levels in rabbit assayed by qRT-PCR. All the results were shown as mean ± SD (*n* = 3). * *p* < 0.05 and ** *p* < 0.01

To investigate the role of CAA and VB6 in TC, we administered CAA combined with high-dosage of VB6 to rabbits in a TC model. Notably, CAA and VB6 treatment could significantly decrease the level of liver enzymes (AST and ALT) compared with TC group (Fig. 1a). In addition, the concentrations of PT, APTT and TT were increased among the coagulopathy rabbits who manifested abnormal coagulation function as well. However, treatment with CAA combined with high-dosage of VB6 alleviated the abnormal expressions of PT, APTT and TT (Table 1). Most importantly, the expression of coagulation factors including II, VII, IX, X and XI was significantly increased after treatment with CAA combined with high-dosage of VB6 compared with control group and TC group (Fig. 1b-d). These results demonstrated that CAA combined with high-dosage of VB6 increased levels of coagulation factors and attenuated TC.

**Table 1:** Coagulation function related indicators

Group	PT (s)	APTT (s)	TT (s)
Sham	9.58 ± 1.14	17.51 ± 2.12	21.51 ± 0.80
TIC	11.92 ± 1.33 **	20.65 ± 2.12 **	23.91 ± 1.57 ***
TIC+AA+VB6	9.52 ± 1.60 **	17.69 ± 2.21 *	22.32 ± 0.92 *

*Note*: *PT* Prothrombin Time, *APTT* Activated Partial Thromboplastin Time, *TT* Thrombin Time

* *P* < 0.05

** *P* < 0.01

***P < 0.001

### CAA combined with high-dosage of VB6 inhibited inflammation and NF-κB signaling pathway in vivo

**Fig. 2:**
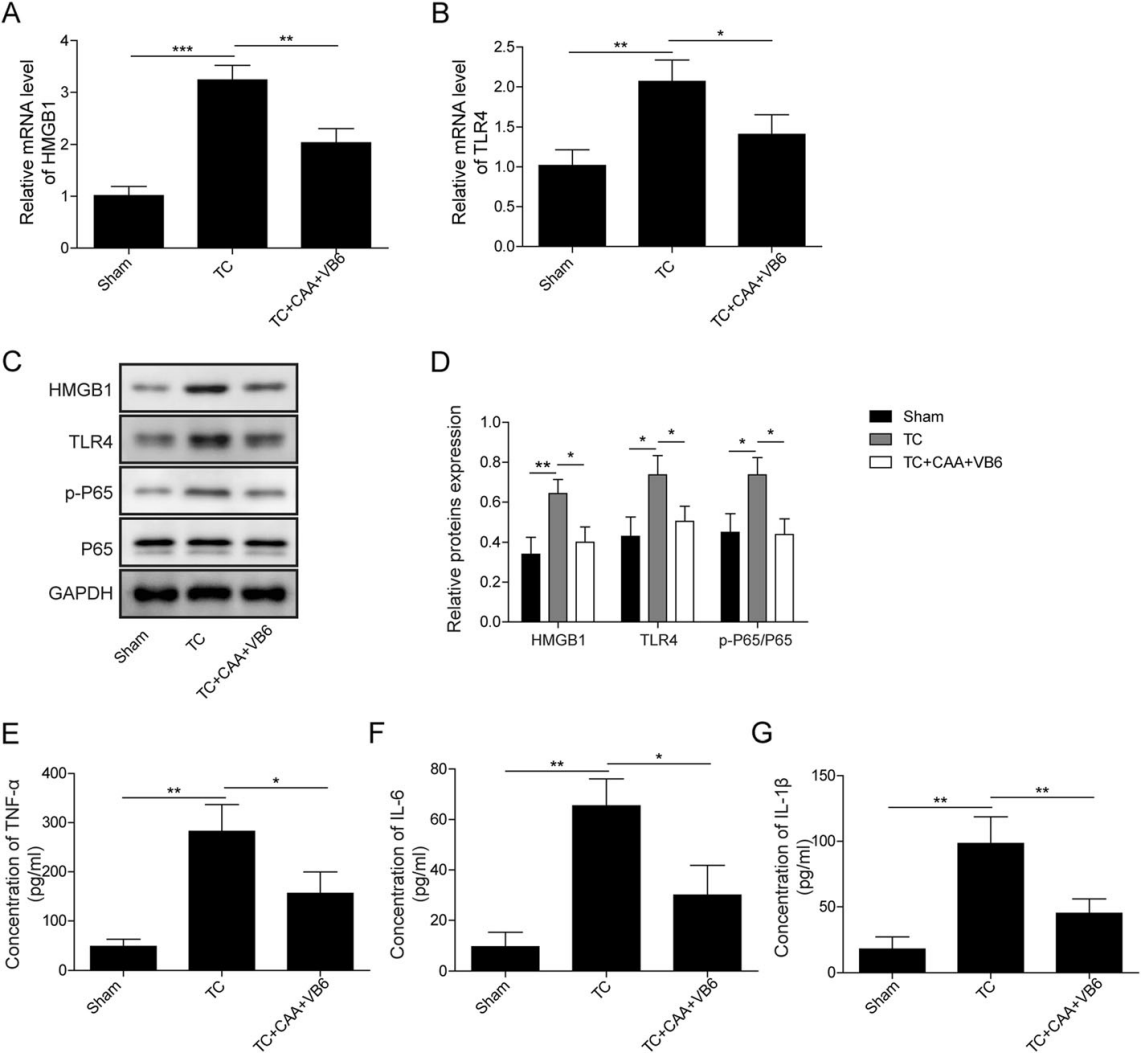
CAA combined with high-dosage of VB6 regulated the expression of HMGB1, TLR4, NF-κB and inflammatory factors in traumatic rabbit’s model. **a&b** The mRNA expression of HMGB1 and TLR4 was detected by qRT-PCR. **c&d** The protein of HMGB1, TLR4, p-P65 and p65 was detected by Western blotting. **e-g** The concentrations of TNF-α, IL-6 and IL-1β were measured by ELISA. All the results were shown as mean ± SD (*n* = 3). * *p* < 0.05, ** *p* < 0.01 and *** *p* < 0.001

We used a rabbit TC model to further study the mechanism of action of CAA combined with VB6 in vivo and the results are shown in Fig. 2. The mRNA level of HMGB1 and TLR4 were increased in the TC group (Fig. 2a&b). However, treatment with CAA combined with high-dosage of VB6 significantly inhibited the mRNA level of HMGB1 and TLR4 (Fig. 2a&b). The protein level changes of HMGB1 and TLR4 were consistent with that of mRNA (Fig. 2c&d). Furthermore, CAA combined with high-dosage of VB6 could inhibit phosphorylation of p65 induced by the trauma, suggesting NF-κB signaling pathway was repressed by CAA combined with high-dosage of VB6 (Fig. 2c&d). Additionally, the significantly increased levels of inflammatory factors such as TNF-α, IL-6, IL-1β appeared in the traumatic group (Fig. 2e-g). However, administration with CAA combined with high-dosage of VB6 inhibited the expression of inflammatory factors (Fig. 2e-g). These results suggested that CAA combined with high-dosage of VB6 alleviates TC by regulating NF-κB signaling pathway and inflammatory factors in vivo.

### CAA combined with high-dosage VB6 repressed endothelial cell inflammation

**Fig. 3:**
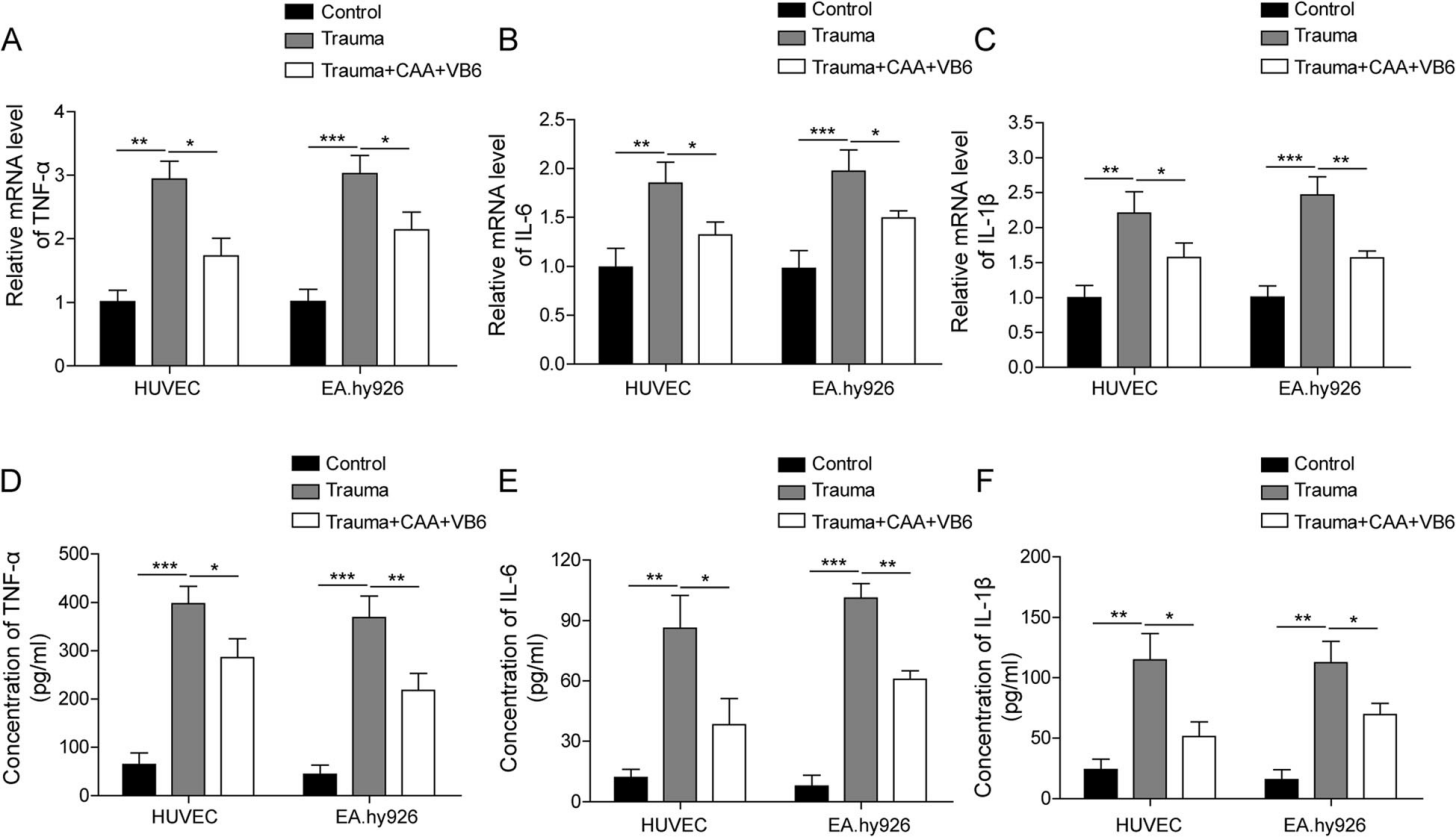
CAA combined with high-dosage of VB6 inhibited endothelial cell inflammatory responses. **a-c** The mRNA expression of TNF-α, IL-6 and IL-1β in HUVEC and EA.hy926 cell was measured by qRT-PCR. **d-f** The release level of TNF-α, IL-6 and IL-1β was detected by ELISA. All the results were shown as mean ± SD (*n* = 3), which were three separate experiments performed in triplicate. * *p* < 0.05, ** *p* < 0.01 and *** *p* < 0.001

To ascertain if CAA combined with high-dosage of VB6 can potentially inhibit endothelial cell inflammation, in vitro cellular injury model in HUVECs and EA.hy926 cells was utilized. The results showed that the levels of TNF-α, IL-6 and IL-1β in the trauma group were obviously elevated compared with control group (Fig. 3a-f). However, compared with the trauma group (Fig. 3a-f), CAA combined with VB6 significantly reduced expression and secretion of these inflammatory factors. These results illustrated that CAA combined with high-dosage of VB6 inhibited endothelial cell inflammatory responses.

### Overexpression of HMGB1 attenuated the effect of CAA combined with high-dosage of VB6 on inflammatory response in HUVECs and EA.hy926 cells

**Fig. 4:**
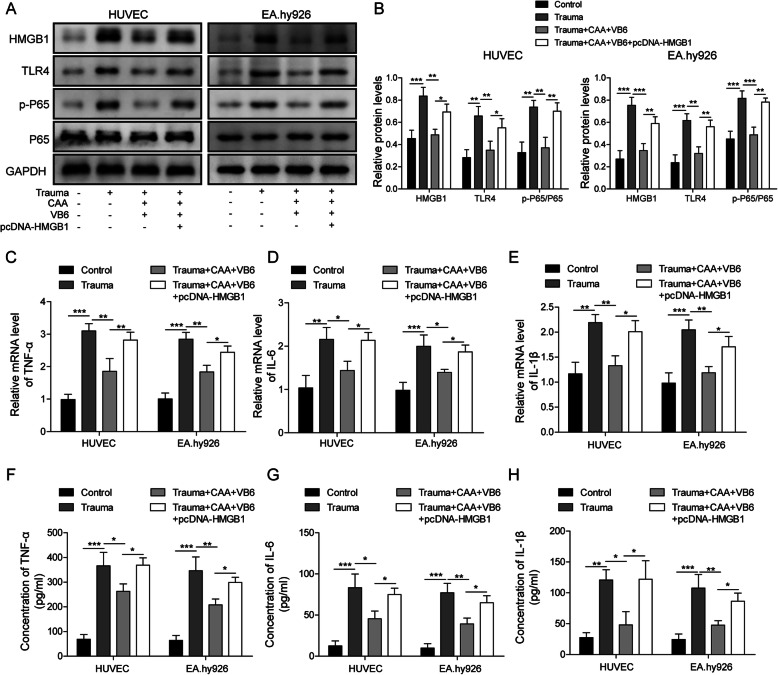
Overexpression of HMGB1 attenuated the effect of CAA combined with high-dosage of VB6 on inflammation in HUVECs and EA.hy926 cells. **a-b** Protein of HMGB1, TLR4 and P65 in cells were detected by Western blotting. The mRNA expression of TNF-α, IL-6 and IL-1β in cells was measured by qRT-PCR **c-e** and the concentration of TNF-α, IL-6 and IL-1β by ELISA **f-h**, respectively. All the results were shown as mean ± SD (*n* = 3), which were three separate experiments performed in triplicate. *  *p* < 0.05, **  *p* < 0.01 and ***  *p* < 0.001

In order to explore the mechanism of action of CAA combined with VB6, the effect of HMGB1 on inflammatory response in vitro was investigated. As shown in Fig. 4a&b, the expression of HMGB1, TLR4 as well as p-p65 was increased in both HUVECs and EA.hy926 cells mediated by trauma, while administration of CAA and VB6 inhibited their (HMGB1, TLR4 and NF-κB) expression. Interestingly, overexpression of HMGB1 reversed the effect of CAA and VB6 (Fig. 4a&b). As shown in Fig. 4c-h, the secretion and mRNA expression of TNF-α, IL-6 and IL-1β were significantly increased in both HUVECs and EA.hy926 cells of the traumatic group. CAA and VB6 intervention significantly reduced these inflammatory factors, but overexpression of HMGB1 could resist this effect (Fig. 4c-h). These findings indicated that CAA combined with VB6 inhibited inflammatory response by regulating the HMGB1-mediated TLR4/NF-κB signaling pathway.

## Discussion

TC remains one of the biggest causes of mortality among trauma patients especially during the acute phase of trauma [22]. Although fluid resuscitation makes the backbone of trauma managements, there is evidence to suggest that it is one of the main drivers of TC [23]. Most importantly, an analysis of hemorrhage related mortality revealed that despite fluid resuscitation interventions, deaths still occur [24]. Therefore, a comprehensive and effective method is needed to treat TC. In present study, a TC model by using HUVECs, EA.hy926 cells and rabbits was established to explore the role of CAA and VB6.

Amino acids, including essential amino acids (EAAs), conditionally essential amino acids (CEAAs) and non-essential amino acids (NEAAs), can promote the expression and processing of defective coagulation factors, increase the expression of anti-inflammatory cytokines, and reduces apoptosis and pro-inflammatory cytokine expression in oxidative stress and inflammation [25, 26]. VB6 is a necessary coenzyme for a variety of enzymes. These enzymes are mainly involved in amino acid metabolism and also participate in lipid and carbohydrate metabolism pathways. Epidemiological evidence from early case-control and prospective studies suggests that low dietary intake or a decrease in blood VB6 concentration increases the risk of cardiovascular disease, but its association with coagulation has not been studied [27]. Liver plays an important role in coagulation, and liver function is important indicators of coagulopathy [28]. The coagulation profile parameters (PT, APTT and TT) provide a diagnostic basis for coagulopathy [29]. Ideally, there is supposed to be elevated expression of liver enzymes and factors during hemorrhage while impaired clotting factors are expected in coagulopathy [30]. Our results indicated that insufficient blood volume can lead to abnormal expression of liver enzymes and coagulation factors, and the treatment of CAA combined with VB6 reversed the factors’ expression trend towards the control group.

Inflammation is one of the causes of advanced TC. It is reported that inflammation can lead to the enlargement of endothelial cell coagulant activation, eventually leading to the production of systemic thrombin and the consumption of coagulation factors and platelets [29]. Additionally, the endothelial cell centered inflammation-coagulation pathway plays an important role in coagulation, ant-coagulation and fibrinolysis in the progress of TC [31]. VB6 was recently reported to inhibit NLRP3 activation and reduce the maturation of caspase-1 and proteolytic maturation of IL-1β to suppress the inflammatory reactions [32]. In present study, the administration of CAA combined with VB6 inhibited the mRNA expression in vitro and the content in cell supernatant of inflammatory cytokines, thereby alleviating TC.

Previous studies on HMGB1 have laid out its significance in the onset or progress of inflammation [33, 34]. Specifically, HMGB1 triggers the TLR4 signaling pathway which then activates NF-κB to induce the expression of inflammatory factors [35]. In addition, studies showed that HMGB1 could be a predictor of coagulopathy, organ failure and related inflammatory responses in severe trauma [36]. Our study found that TC was closely related to the up-regulation of inflammatory cytokines mediated by activation of NF-κB signaling pathway. CAA combined with high-dosage of VB6 could significantly decrease the inflammation and NF-κB signaling pathway. Most importantly, overexpression of HMGB1 effectively reduced the effects of CAA and VB6 by activating TLR4/NF-κB signaling pathway. Taken together, our findings indicated that the HMGB1/TLR4/NF-κB signaling pathway was involved in TC while treated with CAA combined with VB6.

## Conclusion

In conclusion, our study demonstrated a simple but yet an effective way of creating TC model CAA combined with high-dosage of VB6 alleviated TC by inhibiting endothelial cell inflammation by repressing HMGB1-mediated NF-κB signaling pathway. Since TC is one of the leading causes of mortality among trauma patients, the results of this study indicate that high-dose VB6 combined with CAA is a potential therapeutic agent for TC.

## Methods

### Cell culture

Human umbilical vein endothelial cell (HUVEC) and EA.hy926 cell were purchased from the Cell Bank of the Chinese Academy of Science (Shanghai, China). Cells were cultured in endothelial cell growth medium (Gibco, Life technologies, USA) containing 10% FBS, 20 μg/mL VEGF (Gibco), and 1% penicillin/streptomycin. The cells were cultured in an incubator at 37 °C with a humidified atmosphere of 5% CO_2_.

### Establishment of cellular inflammation model and treatment of CAA and VB6

HUVEC and EA.hy926 cell monolayers were established in the microfluidic channels of a microfluidic device well plate, which was perfused at constant shear overnight. Cells were then exposed to epinephrine at 37 °C with 5% CO_2_, 95% N_2_ for hypoxia up to 12 h [37]. The cells were then transferred to fresh medium and incubated at 37 °C with 5% CO_2_ incubator for reoxygenation for 2 h [38]. 1 mmol of pyridoxine and 85 mg/ml of CAA were used to treat HUVECs and EA.hy926 cells.

### Cell transfection

The HMGB1 full length was cloned into pcDNA3.1 overexpression plasmid. The cells (5 × 10^5^) were planted in 6-well plates 24 h prior to transfection with pcDNA3.1-HMGB1 with 60–70% confluence, then transfected using Lipofectamine 2000 (Invitrogen, USA). Cells were harvested at 48 h for further experiments.

### Rabbit TC model construction

Rabbits were chosen in the in vivo model in order to increase the operational stability and the similarity of the disease model and human diseases [39–42]. 15 female New Zealand white rabbits (5 per group, age: 2–3 month) were purchased from Shanghai SLAC Laboratory Animal Co. Ltd. (Shanghai, China). Throughout the study, all rabbits were housed at 23–25 °C and 50% humidity on a 12 h/12 h light-darkness cycle. They were housed at one rabbit per cage and fed on rabbit pellets while tap water was provided ad libitum. All study protocols followed recommended guidelines on animal handling and were approved by the Organizational Ethics Committee.

A model of TC dysfunction was established then. After normal rabbits (2.5–3.0 kg) were anesthetized, trauma (laparotomy) and foot artery bloodletting was maintained to maintain blood pressure of 40-50 mmHg or 40–50 mmHg (200 ml). After 1% maintenance, an animal model of TC was made. The experiments were divided into control group, trauma-induced coagulopathy (TIC) group and treatment group. The control group was a normal animal without any treatment. The treatment group was given 40 ml of CAA and 16 ml of VB6 injection. And 3 ml blood sample was taken from the ear margin vein for testing. The liver tissues were cut into small pieces and quickly store at − 80 °C until use.

### AST and ALT assay

EnzyChrom™ alanine aminotransferase (ALT) assay kit and EnzyChrom™ aspartate aminotransferase (AST) assay kit (Bioassay System, Hayward, California) were used to measure ALT and AST activity as indicators of liver damage.

### Coagulation function assay

The blood sample extracted from rabbit traumatic coagulopathy model was transferred into 1.5 mL tube with 3.8% sodium citrate. The blood was then centrifuged at 1000 rpm for 15 min at 4 °C to collect plasma. The concentrations of activated partial phromboplastin time (APTT), prothrombin time (PT) and thrombin time (TT) were measured by using commercial diagnostic kits according to the manufacturers’ instructions. The detection was done within 3 h after blood collection.

### qRT-PCR assay

Total RNA were extracted from HUVEC, EA.hy926 cell and rabbit’s plasma upon treatment completion using TRIzol. The RNA extraction procedure was performed accordingly to manufacturer’s protocol. Following successful extraction, the total RNA was reverse transcribed using the PrimeScript RT reagent Kit (Takara). The resultant cDNA was used for qRT-PCR reactions which were performed using SYBR-Green Master Mix (Takara) at a total volume 10 μl, comprised of 100 ng cDNA as template, 0.2 μM each primer, and 1 × SYBR-Green Master Mix. The amplification procedure was as follow: Initial denaturation at 95 °C for 30 s, next performed 40 cycles of 95 °C for 5 s, 58 °C for 15 s and 72 °C for 15 s, and a final extension at 94 °C for 15 s. The target gene expression data was analyzed with the 2^-ΔΔCq^ method [19]. The primer sequences used were as follows: 5′-ATATGGCAAAAGCGGACAAG-3′ (forward) and 5′-GCAACATCACCAATGGACAG-3′ (reverse) for HMGB1; 5′-AGAAACTGCTCGGTCAGACG-3′ (forward) and 5′- AATGGAATCGGGGTGAAGGG − 3′ (reverse) for TLR4; 5′-AGTCCGGGCAGGTCTACTTT-3′ (forward) and 5′-GGCCACTACTTCAGCGTCTC-3′ (reverse) for TNF-α; 5′-GTCCGGAGAGGAGACTTCAC-3′ (forward) and 5′-ACAGTGCATCATCGCTGTTC-3′ (reverse) for IL-6; 5′-ACAGCAATGGTCGGGACATA-3′ (forward) and 5′-TGAGAGACCTGACTTGGCAG-3′ (reverse) for IL-1β; 5′-CCAGGTGGTCTCCTCTGA-3′ (forward) and 5′-GCTGTAGCCAAATCGTTGT-3′ (reverse) for GAPDH.

### Inflammatory cytokines assay

The cell suspension was centrifuged at 1500 g for 15 min at room temperature to collect the supernatant. ELISA kits (Abcam, Cambridge, Massachusetts) were used to determine inflammatory factor (IL-6, TNF-α, and IL-1β) levels according to the protocol instructions.

### Western blot assay

Use RIPA Lysis Buffer (Beyotime, Shanghai, China) to lyse cell samples. Note that PMSF (Amresco, Houston, Texas, USA) is added before lysis. The content of lysed protein was determined using a BCA protein assay kit. Protein was electrophoresed on a 10% sodium lauryl sulfate polyacrylamide gel (SDS-PAGE) and then transferred to a polyvinylidene fluoride (PVDF) membrane. HMGB1 (ab18256; Abcam), TLR4 (ab13867; Abcam), p65 (#8482, Cell Signaling Technology), p-p65 (#3033, Cell Signaling Technology) and GAPDH (ab181602; Abcam) primary antibodies were added and incubated overnight at 4 °C. Following washing with TBST, the horseradish peroxidase-labeled secondary antibody and the PVDF membrane(s) were incubated for 2 h at room temperature. The chemiluminescence detection kit (Millipore) and a gel imaging were used to visualize protein bands.

### Statistical analysis

All data were statistically analyzed using GraphPad Prism 6.0 software and are expressed as the mean ± standard deviation (SD). Students’ t-test was used to compare the difference between two groups. One-way analysis of variance (ANOVA) followed by Tukey post hoc test was used for multiple comparisons. *P* < 0.05 was considered significant difference.

## Data Availability

All data generated or analysed during this study are included in this published article [and its supplementary information files].

## Abbreviations

ALT: Alanine aminotransferase
APTT: Activated partial thromboplastin time
AST: Aspartate aminotransferase
CAA: Compound amino acid
HMGB1: High-mobility group box 1
HUVEC: Human umbilical vein endothelial cells
IL-1β: Interleukin-1β
IL-6: Interlukin-6
NF-κB: Nuclear Factor Kappa B
PL: Pyridoxal
PM: Pyridoxamine
PN: Pyridoxine
PT: Prothrombin time
TC: Traumatic coagulopathy
TLR4: Toll like receptor 4
TNF-a: Tumor necrosis factor- a
TNF-α: Tumor Necrosis Factor-α
TT: Thrombin time
VB6: Vitamin B6
